# Heat Management in Thermoelectric Power Generators

**DOI:** 10.1038/srep20951

**Published:** 2016-04-01

**Authors:** M. Zebarjadi

**Affiliations:** 1 Department of Mechanical and Aerospace Engineering, Rutgers University, Piscataway, New Jersey, 08854, USA; 2 Institute of Advanced Materials, Devices, and Nanotechnology, Rutgers University, Piscataway, NJ, 08854, USA

## Abstract

Thermoelectric power generators are used to convert heat into electricity. Like any other heat engine, the performance of a thermoelectric generator increases as the temperature difference on the sides increases. It is generally assumed that as more heat is forced through the thermoelectric legs, their performance increases. Therefore, insulations are typically used to minimize the heat losses and to confine the heat transport through the thermoelectric legs. In this paper we show that to some extend it is beneficial to purposely open heat loss channels in order to establish a larger temperature gradient and therefore to increase the overall efficiency and achieve larger electric power output. We define a modified Biot number (Bi) as an indicator of requirements for sidewall insulation. We show cooling from sidewalls increases the efficiency for Bi values less than one, and decreases the efficiency for Bi values larger than one.

Thermoelectric power generators are proposed for many applications such as waste heat recovery (as topping cycles)^1^,^2^,^3^,^4^, automobile industry, and solar thermoelectric power generators^5^,^6^,^7^. It has been shown that the performance of the thermoelectric power generators is an increasing function of its material figure of merit, 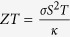, where 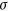 is the electrical conductivity, S is the Seebeck coefficient, T is the operating temperature and 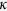 is the thermal conducitity^2^. Typical commercial thermoelectric devices are made out of Bi_2_Te_3_/Sb_2_Te_3_ for room temperature applications and PbTe for high temperature applications and they have a ZT of around 1. These modules are made out of many p-n legs, which are placed thermally in parallel and electrically in series. As an example, HZ-14 model developed by Hi-Z Company, has 6.27 cm by 6.27 cm ceramic area with 49 p-n pairs of bismuth telluride based semiconductors and a thickness of about 5 mm^8^. The module provides 25 W (5% efficiency) output for a temperature difference of 300 °C. This is equivalent of 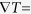
60 °C/mm which is quite large. According to the Goldsmid analysis^9^, the ideal efficiency of the thermoelectric module used in this example (i.e. for a ZT ~ 1 and the temperature difference of 300 °C) is about 10%. Clearly, there is a factor-of-two difference between ideal and practical efficiency, which could be attributed to thermal/electrical contact resistances, and non-ideal heat managements (operational conditions). Both issues have been largely studied in the literature in the past. In this work, we would like to address the heat management issue. It has been shown by several authors, that heat loss through sidewalls of thermoelectric legs, result in lowering of the thermoelectric efficiency^10^,^11^,^12^. The argument here is simple. Opening of heat loss channels, results in less conversion of heat to electricity and therefore lowering the thermoelectric efficiency, a direct conclusion. The purpose of this article is to point to practical issues resulting in cases, wherein opening of heat loss channels, improves the efficiency!

## Methods

**Figure 1:**
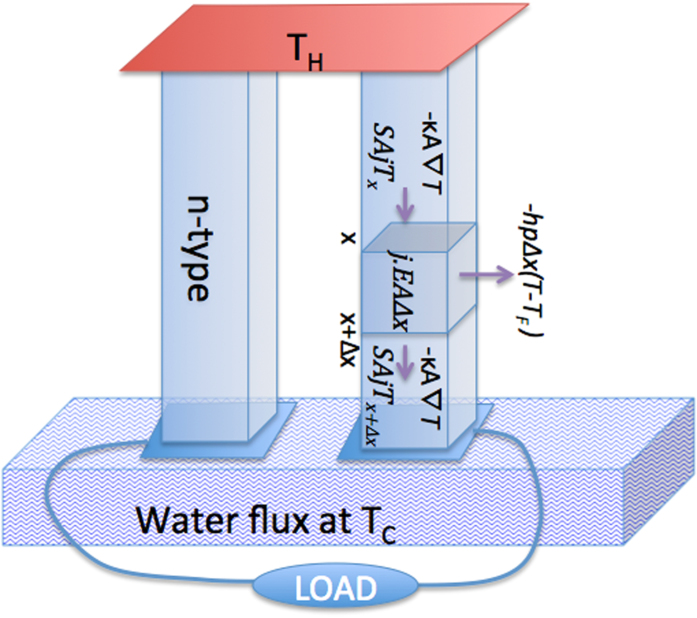
Schematic diagram representing a single n-p pair thermoelectric module. The module is in well contact with a heat source (no interfacial resistance). From the other end, it is cooled down by a liquid flux. Energy balance over a slab of p leg is shown and heat losses through sidewalls are accounted for using convective heat transfer.

Consider a single n-p thermoelectric module schematically shown in Fig. 1. We assume constant materials properties in each leg. 
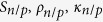
 are the Seebeck coefficient, the electrical resistivity and the thermal conductivity of the n/p legs respectively. The n-p legs are connected electrically in series and thermally in parallel. Therefore the electrical resistance, R, and the thermal conductance, K, of a single n-p pair, ignoring the metallic connections and ignoring the interfacial resistances, could be written as: 
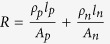
 and 
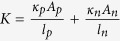
 (this is the total thermal conductance and has the contribution of electrons and phonons). The Seebeck coefficient of the n-p pair is 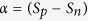 and finally, figure of merit is defined as 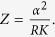


In a pioneer work, Altenkirch^13^/Ioffe^1^ developed an analytical model with constant materials properties to address the efficiency of thermoelectric power generators. The model is later re-formulated by Goldsmid^14^, wherein he assumed one dimensional transport within the thermoelectric legs, neglecting convective heat loss from the perimeter (perfect insulation) and also neglecting contacts. He applied constant temperature boundary conditions (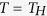 at hot side and 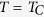 at cold heat sink), and proved that the maximum achievable efficiency (for optimum external load) could be written in terms of n-p figure of merit (Z) and the temperature difference (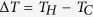) as:


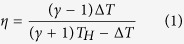



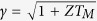



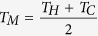


It is clear from Eq. 1 that larger Z values and larger temperature differences result in higher efficiencies. Consequently, the natural tendency is to (1) insulate thermoelectric legs to minimize heat loss and run the module as close as possible to the ideal conditions (perfect insulation) and (2) impose a large temperature gradient by connecting one end to a heat source with large heat fluxes and high temperatures, and cooling down the other end by cold air/water fluxes. There is no doubt that such approach is correct if we assume temperature of the hot/cold ends (of the thermoelectric legs) are exactly the same as the hot heat source/cold heat sink. In practice this is not the case. There is always a temperature drop at the interfaces of the heat source/hot TE end and heat sink/cold TE end. This temperature drop is outside of the thermoelectric leg and does not result in TE power generation. If the cold end is cooled down by a fluid flux at temperature 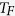, the temperature at the cold end of the thermoelectric leg is not equal to and is larger than the fluid temperature 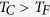. The correct boundary condition in this case, is matching the heat flux at the cold side; to the convective heat transfer flux from the thermoelectric leg to the fluid (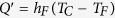). Only when the heat transfer coefficient of the fluid goes to infinity (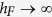), 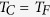 and constant temperature boundary conditions can be used. In many cases, cooling of the cold side is too expensive and thermoelectric modules are simply attached to a heat source and the cold end is cooled by natural convection for which, 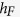
is only about 1 W/m^2^K. In the case of forced air convection (using a fan), 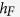
can increase to about 100 W/m^2^K. Water-cooling is more expensive but it can increase the heat transfer coefficient to quite large values (10–1000 W/m^2^K) and to even larger values when forced water-cooling is used.

In cases wherein poor cooling is performed, a natural consequence of sending a large heat flux to the thermoelectric modules, is over heating of the module and consequently establishment of much smaller temperature differences (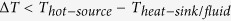) which therefore lowers the overall efficiency. In such cases, it might be possible to increase the temperature difference simply by opening heat loss channels at sidewalls. That is to remove the insulation layers and allow a larger surface area to be in contact with the cooling source to establish a larger temperature difference along the leg. The main question is what is the best operating conditions in which efficiency is large enough with cheaper cooling options.

To answer this question, we develop a more realistic model considering convective heat transfer loss from the perimeter and considering a more realistic convective boundary condition at the cold side. Only if the heat transfer coefficient goes to infinity, the boundary condition at the cold side is 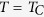. The geometry and the detailed derivation of the heat conduction equation are shown in Fig. 1.

Here to simplify we only write the equation for the p leg and therefore drop the sub-index p for materials/geometrical properties. The results could be simply extended to include both types by defining total thermal/electrical resistances of the n-p legs. The results of writing the heat balance equation, as shown in Fig. 1, and as discussed in our previous publication^15^, is a heat conduction equation for electrons and phonons combined.


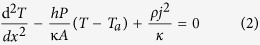


and the heat flux is defined as:


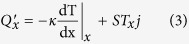



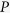
 is the leg perimeter, A is the cross section of the leg, 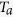 is the ambient temperature, 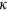 is the total thermal conductivity due to electrons and phonons, and 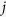 is the electric current flux. Note that in Eq. 2, we are neglecting the Thomson effect to simplify the solutions. In other words, we assume that the Seebeck coefficient is not changing with temperature (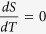). To include the temperature dependent Seebeck coefficient, Thomson term should be added to left hand side of Eq. 2 as – 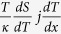. Full solutions with inclusion of Thomson terms, are too lengthy and it is hard to extract information from such complicated equations^12^,^16^.

To further simplify the solutions, we define a set of dimensionless parameters and we also change the reference for measuring the temperature:


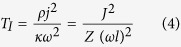



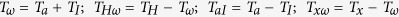



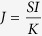



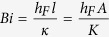



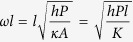


where *I* is current (in units of Amp), 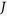 is a dimensionless current. 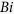 is similar to the Biot number, defined at the cold end with 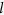 being the length of the thermoelectric leg and 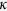, the thermal conductivity of the TE module. 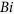 is representative of the effectiveness of cooling at the cold side. Larger 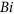 values correspond to larger 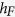 values and therefore better cooling at the cold end. 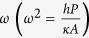 could be viewed as damping term, temperature drop along the TE legs is proportional to 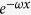. 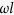 is another dimensionless parameter, which is known as the fin parameter. In this analysis, it is the representative of heat loss through sidewalls and increases as 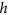 (the heat transfer coefficient through the sidewalls) increases. The main approximation in our model is that temperature is constant in the y-z plane. Note that this assumption is used very often in modeling of fins and is valid for fin parameters smaller than 3^17^,^18^.

The solution of Eq. 2, for fixed temperature boundary condition (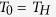) at the hot side and convective boundary conditions at the cold side 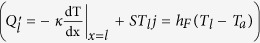 is


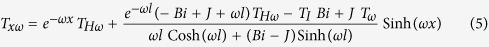


Note that here to simplify the problem; we assumed that the temperature of the cooling fluid (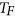) is the same as the ambient temperature (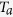). The difference between the cooling of the cold side and the heat loss through the sidewalls is shown with the two different heat transfer coefficients 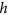 and 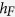.

Using Eq. 3, the heat rate at the hot side (x = 0) is:


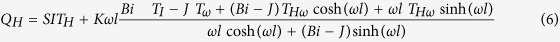


It can be shown that the useful work done on the external load is:


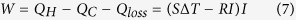



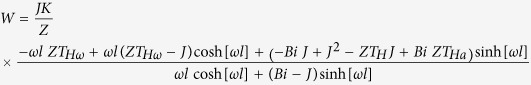


Finally the efficiency is


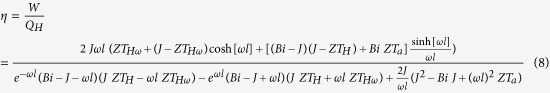


## Results

**Figure 2:**
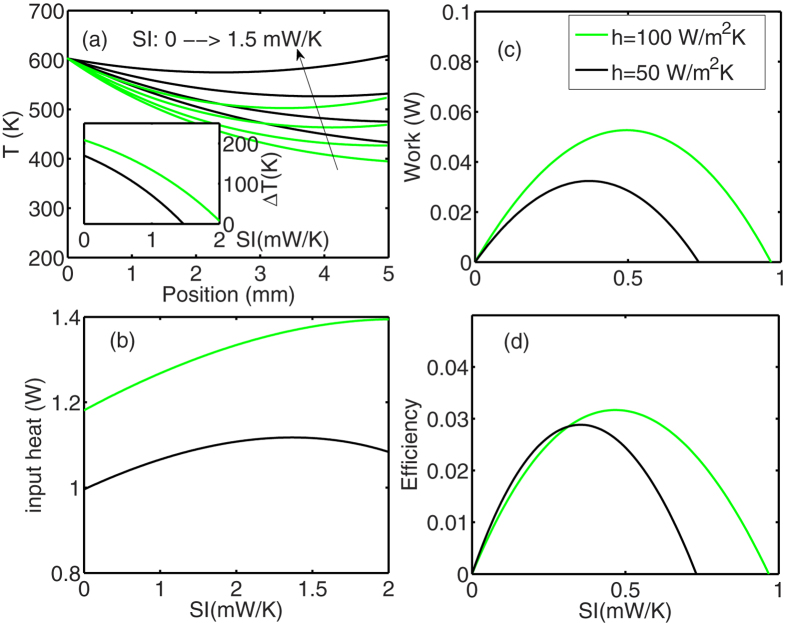
Results for a single p-n pair, the dimensions are 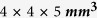, 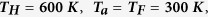 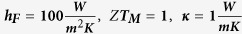. Black lines are plotted for 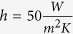 and green lines for 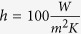. (**a**) Temperature distribution along the thermoelectric leg for different SI values which is increased from 0 to 1.5 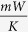 with steps of 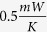
and using Eq. 5. Inset: 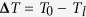 as a function of SI (in 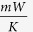
units). (**b**) Input heat current (heat current at the hot side, 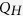, Eq. 6) as a function of SI, (**c**) Useful output work (Eq. 7) as a function of SI and (**d**) Efficiency of heat to electricity conversion (Eq. 8) as a function of SI.

To look at a practical device performance, we take dimensions from a typical thermoelectric module. For example those of the HZ-14 discussed earlier in the introduction and is summarized in the caption of Fig. 2.

Figure 2a shows the temperature distribution along thermoelectric legs, when the hot side is kept at 600 K, and the cold side is cooled down by a coolant fluid of temperature 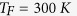
and heat transfer coefficient of 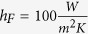. Convective heat loss from sidewalls is also considered, assuming that the walls are in contact with a fluid at ambient temperature, which is taken to be the same as the coolant temperature 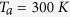, but with a different heat transfer coefficient (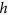). Graphs for different applied currents and two different 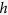 values are plotted. To prevent any prior assumption on the Seebeck coefficient, instead of current we use SI (Seebeck times current), which could be tuned by tuning the electrical current through the external load. As current increases, the temperature difference on the sides of the TE leg decreases as a result of Joule heating. This is shown in the inset of Fig. 1a. The input heat pumped at the hot side (Q_H_) is plotted in Fig. 2b and increases as more current is passed (as current carries more heat into the leg). Finally there is an optimum work and efficiency versus current (Fig. 2c,d). Figure 2 is plotted for two different sidewall heat transfer coefficients. Even at this relatively large coolant heat transfer coefficient (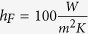), we can clearly see that increasing the heat loss through the sidewalls (i.e. increasing 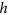 from 50 to 100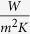), results in establishment of larger temperature differences and therefore larger efficiencies as shown in Fig. 2d.

**Figure 3:**
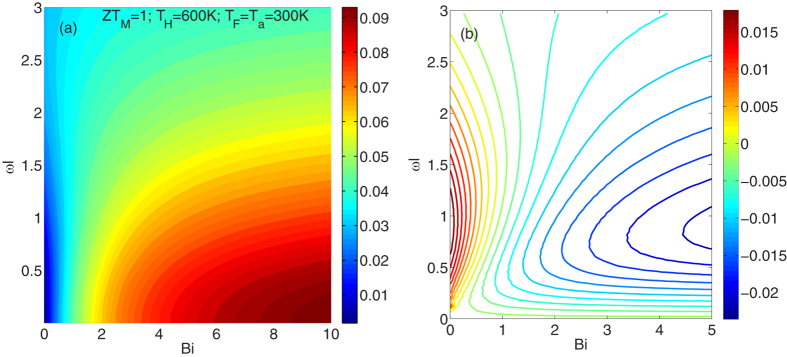
(**a**) Optimum efficiency versus 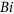, and 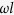, plotted for 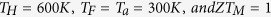. Ideal efficiency (from Eq. 1) for the set of chosen parameters is 10%. (**b**) The derivative of efficiency with respect to 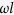 using the same parameters as in part (**a**).

We know at the limit of 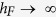, the results will be completely different and larger 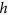 values, result in more heat loss and less efficiency. So the next natural question is that how large is large enough to be considered as the limit of infinity and when we will switch from gaining by opening heat loss channels to loosing by opening such channels. To answer this question, we note that for a fixed set of temperatures, efficiency is a function of 4 different parameters namely Z, J, 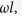
and 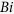 (see Eq. 8). Among these parameters, J is irrelevant in the sense that it is tunable and for a given thermoelectric module, we can optimize the efficiency versus this parameter. Z parameter could be fixed, as we know good commercial thermoelectric materials possess 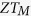 values close to 1 and in the research labs this value is close to 2. The remaining two parameters, 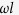
and 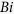, are then the crucial parameters in our discussion. To find the transition point, we fixed the temperatures and the figure of merit. Then, for a given 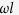
and 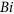, we optimized the efficiency versus J. The resulted optimum efficiency is plotted in Fig. 3. For the set of chosen parameters, the ideal efficiency, according to the Goldsmid analysis, Eq. 1, is about 10%. Fig. 3 shows that this ideal efficiency is only achievable for 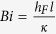 values larger than 8. For a thermal conductivity of 1 W/mK, and length of 5 mm, this corresponds to 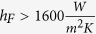. In this regime, as expected, any heat loss (increase of 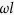) results in lowering of the efficiency. The transition happens at around 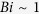. Below this value, the efficiency increases as heat loss through sidewalls increases (when 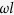 increases), which is counter-intuitive and is happening due to the consequent establishment of a larger temperature difference. The transition point could be more easily identified from Fig. 3b. In this figure the derivative of the efficiency with respect to 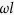 is plotted. This derivative is positive for small Bi values and is negative for larger ones. The transition happens around 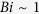. The absolute values of efficiency are sensitive to the chosen temperatures (of the hot side and of the ambient/fluid) and the Z parameter. However, the overall shape of this graph (Fig. 3) has only minor sensitivity to any of these parameters and therefore the 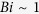 value could be taken as the transition value independent of the Z and the temperature values. In fact, the overall result of increasing Z parameter and temperature difference is to shift the transition point slightly to larger values. Which means for larger ZT values, even larger 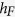 values are required for efficient heat conversion. The studied case of Fig. 2 was corresponding to 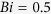 which is clearly in the regime wherein heat loss through sidewalls is beneficial.

Finally, we would like to address the issue of thermal interfacial resistance, which was ignored in our calculations. The thermal interfacial resistance of dissimilar interfaces is on the order of 10
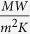
19. This number should be compared to the thermal conductance per unit area of the thermoelectric leg. For the studied case, 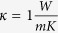 and TE length of 5 mm, the thermal conductance per unit area is 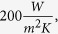 which is orders of magnitude smaller than the interfacial conductance. This means thermal interface resistance could be ignored. In extreme cases, where there is considerable effect from the interface, its effect could be added to modify and reduce the thermal conductivity of the TE leg effectively. This effective thermal conductivity will modify the Bi and 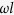 numbers accordingly, but the conclusions do not change. The reader should note that in this work, we only considered the effect of poor cooling and we assumed that the hot side is properly connected to a high heat flux. Considering the low efficiency of the thermoelectric modules, cases with poor heating and poor cooling do not result in meaningful power outputs.

In summary, we developed a fin model for thermoelectric power generators, including the convective heat transfer coefficient from the sidewalls. We determined that in addition to the Z parameter and the temperatures, the device efficiency is also a function of two dimensionless parameters. One is 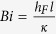 (Biot number) which represents the effectiveness of the cooling system used through 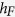 value and the other is 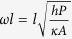 (fin parameter) which represents the heat loss through sidewalls. We showed that if 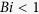, that is when poor cooling is used at the cold side, opening heat loss channels through the sidewalls, increases the overall efficiency of the thermoelectric module as a result of formation of a larger temperature difference over the thermoelectric legs. In the opposite regime, when 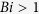, we recovered the standard predicted behavior of thermoelectrics, wherein extra heat loss results in lowing of the heat to electricity conversion efficiency.

## Additional Information

**How to cite this article**: Zebarjadi, M. Heat Management in Thermoelectric Power Generators. *Sci. Rep.* **6**, 20951; doi: 10.1038/srep20951 (2016).
